# Successful Treatment of Liver Aspergilloma by Caspofungin Acetate First-Line Therapy in a Non-Immunocompromised Patient

**DOI:** 10.3390/ijms130911063

**Published:** 2012-09-06

**Authors:** Qing-Xian Bai, Yi Huan, Jian-Hong Wang, Li-Jie Yang, Hong-Juan Dong

**Affiliations:** 1 Department of Hematology, State Allogeneic Hematopoietic Stem Cell Transplantation Center, Xijing Hospital, Fourth Military Medical University, Xi’an 710032, China; E-Mails: wangjh_2@163.com (J.-H.W.); yanglj_xj@126.com (L.-J.Y.); dhongjuanyahoo@163.com (H.-J.D.); 2 Department of Radiology and Medical Imaging Center, Xijing Hospital, Fourth Military Medical University, Xi’an 710032, China; E-Mail: huanyi@fmmu.edu.cn

**Keywords:** aspergillosis, liver, *Aspergillus fumigatus*, caspofungin acetate

## Abstract

Aspergillosis remains to be a life-threatening complication in immunocompromised patients. However, *Aspergillus* infection can be observed in non-immunocompromised individuals in rare cases. We report a case of liver aspergilloma in a chronic aplastic anemia patient under relatively intact immune status. Therapeutic strategy for this rare condition was extensively discussed and caspofungin acetate single agent first-line therapy was applied after careful consideration. Encouraging clinical and radiologic improvements were achieved in response to the antifungal salvage. Our long-term follow-up study also revealed a favorable prognosis. Based on this experience, we suggest caspofungin acetate as first-line therapy for treatment plans of liver aspergilloma.

## 1. Introduction

*Aspergillus* is ubiquitously distributed in the environment and its spores can be easily inhaled and develop aspergillosis under certain conditions. Aspergillosis, in particular invasive aspergillosis, remains a serious and lethal complication in immunocompromised patients who have received extensive chemotherapy, immunosuppressants or have undergone solid organ and stem cell transplantation, reaching approximately 80% mortality [1].

*Aspergillus* infection can be systemic or local, depending on the immune defense against these fungi. Among nearly 185 species of *Aspergillus*, *Aspergillus fumigatus* (*A. fumigatus*) is the most prevalent pathogen and accounts for more than 90% of all infections [2]. Clinical symptoms, including fatigue, weight loss and low-grade fevers, may be present for weeks or months prior to a correct diagnosis, and often mimic the manifestations of malignancies or infections. *A. fumigatus* predominantly affects lungs and the naso-orbital sinus. Other unfavorable organs such as gastrointestinal, cutaneous, cardiovascular, and central nervous system can be involved in immunocompromised patients [3,4]. Of special interest, recent studies have highlighted that *A. fumigatus* might affect non-immunocompromised hosts on rare occasions [5].

## 2. Case Presentation

A 55-year-old woman with progressive hepatic region discomfort was referred to a hematological department in May 2006. Her previous medical history was significant for chronic aplastic anemia and she was treated with stanozolol and intermittent γ-globulin infusion. Full blood and bone marrow examination on a regular time schedule showed significant remission.

**Figure 1:**
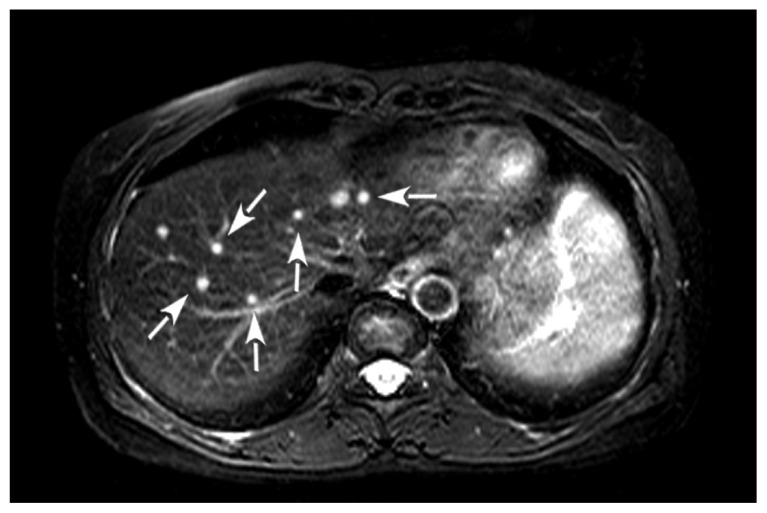
Horizontal abdominal MRI image in May 2006 shows multiple solid nodules in the right lobe of the liver (arrows indicated).

On admission, the patient had a body temperature of 36.5 °C, pulse of 75 beats per minute, blood pressure of 120/80 mmHg, respiratory rate of 16 per minute, and oxygen saturation of 98% on room air. Her abdomen was soft to palpation. Full blood test showed leukocyte count of 3.0 × 10^9^/L, hemoglobin 110 g/L and platelet 23 × 10^9^/L. Immunological studies, including quantitative immunoglobulins analysis, CD4/CD8 T lymphocyte ratio, and delayed hypersensitivity skin tests, were all normal. No predisposing disease associated with immunosuppression, such as diabetes mellitus, was found. Her human immunodeficiency virus status was negative, indicating the patient was in a non-immunocompromised condition. Abdominal ultrasonography and magnetic resonance imaging (MRI) showed multiple heterogeneous solid nodules in the right lobe of the liver (Figure 1). No abdominal lymphadenopathy or effusions were visible.

Malignant ailments and metastatic diseases were initially suspected, but serum tumor marker screening (including CEA, CA-125, CA-199, PSA, AFP, *etc*.) was within normal limits. Her aspartate aminotransferase (AST) was 80 U/L (normal range <40 U/L) and alanine aminotransferase (ALT) was 65 U/L (normal range <40 U/L). Other laboratory investigations, including erythrocyte sedimentation rate (ESR), C-reactive protein (CRP), galactomannan antigenemia (GM) test, and autologous antibody, performed twice a week, were not specific. Complete liver disease studies, including hepatitis virology, cytomegalovirus, Epstein-Barr virus serology, urinary copper, serum ceruloplasmine failed to lead a significant result.

**Figure 2:**
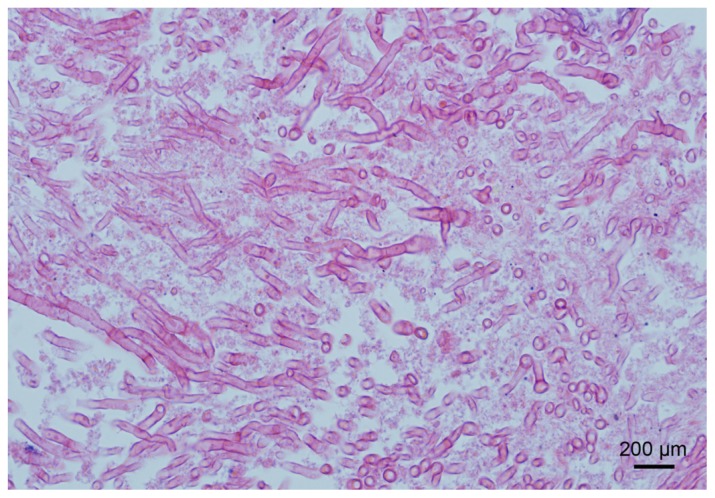
Profound *Aspergillus* hyphae were observed in the necrotic liver specimen. Magnification at 400×, bar = 200 μm.

Two weeks after admission, ultrasound-guided fine needle aspiration was performed. On histopathological examination, abundant fungal hyphae were observed in the background of necrosis and chronic inflammation (Figure 2). *A. fumigatus* was subsequently isolated and cultured from the biopsy aspirate. Bacterial and acid-fast smears and cultures were negative. Pulmonary aspergillosis with liver dissemination was suspected, but the patient denied relevant infectious and occupational exposure history. A comprehensive whole body evaluation, including chest and paranasal sinus computed tomography (CT), did not indicated aspergillosis lesions. Regular GM tests continued to be negative. On the basis of these findings, we concluded the diagnosis of liver aspergilloma.

**Figure 3:**
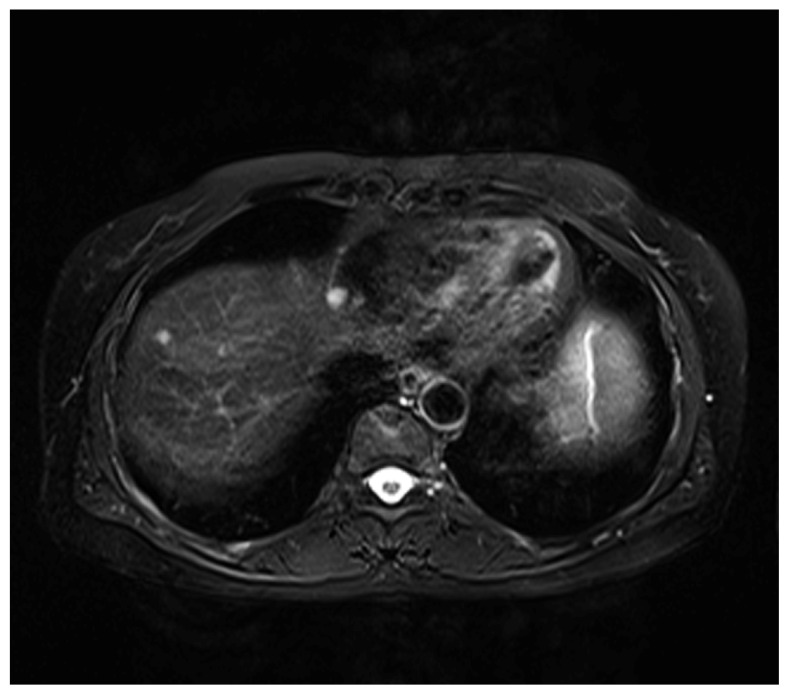
After receiving two courses of caspofungin acetate first-line therapy, follow-up horizontal abdominal MRI image showed evident remission.

The patient was prescribed caspofungin acetate (Cancidas^®^, Merck Sharp & Dohme Pty. Ltd., Australia) according to the minimal inhibitory concentrations (MICs) tests. An antifungal regimen was started with caspofungin acetate 70 mg on day 1 and 50 mg daily from day 2 to day 10. Serum liver enzymes were monitored to interrupt potential adverse effects. The patient received one course of caspofungin acetate first-line therapy every month and responded well in the clinical symptoms. Two months after the initial diagnosis, repeated MRI images showed a significant reduction in the sizes and number of the liver nodules (Figure 3). Our patient underwent caspofungin acetate therapy for six months and was discharged. During our last time follow-up in May 2012, she was stable without signs of progression or recurrence.

## 3. Discussion

This case is interesting because the radiological findings are not typical for liver aspergilloma and the therapeutic plans of caspofungin acetate single agent first-line therapy have not yet been reported.

The etiology of aplastic anemia is considered to be an immune-mediated bone marrow failure and its therapeutic strategy usually involves immunodepressants [6]. Such medical agents include anti-thymocyte globulin (ATG), anti-lymphocyte globulin (ALG), and cyclosporine. Aplastic anemia patients who receive these medications are at a higher risk of developing invasive aspergillosis because of extensive immune suppression [7,8]. Our patient had refused immunodepressants and her regimens were stanozolol and intermittent γ-globulin infusion, which maintained her in a relatively non-immunocompromised condition.

Distinctly different from the typical pattern of invasive aspergillosis in immunocompromised patients, increasing numbers of studies suggest aspergilloma predominantly occurs in non-immunocompromised hosts [5]. *Aspergillus* has angioinvasive properties and frequently disseminate from the primary lesions, usually the lung, to a variety of organs via hematogenous spread. According to a retrospective study, the liver can be involved in about 15% cases of aspergillosis, but is almost always followed by dissemination from the lung [9]. The present case described a fortuitous discovery of liver aspergilloma without lung aspergillosis in a non-immunocompromised patient. Similar to our findings, there are three cases [10–12] of primary liver aspergillosis in immunocompromised hosts during the past decade (Table 1). However, our patient was in a relatively non-immunocompromised condition and *Aspergillus* infection was restricted to the liver. To the best of the authors’ knowledge, this is the first case of liver aspergilloma without dissemination over the past ten years, in particular in a non-immunocompromised host.

Therapeutic decisions for aspergilloma are controversial. Some experts suggest surgical resection combined with antifungal drugs while others recommend antifungal salvage [13–16]. Being aware that the aspergilloma was multiple, surgical resection was not arranged. Voriconazole (azoles) and caspofungin acetate (echinocandins) were new generations of broader spectrum antifungal regimens recommended as front-line therapeutic agents for invasive and refractory aspergillosis [17–19]. MICs tests showed the *A. fumigatus* species was sensitive to voriconazole and caspofungin acetate but resistant to other antifungal agents, including fluconazole and itraconazole. Moreover, inspiring outcomes have been documented by using voriconazole in combination with caspofungin acetate [20–23]. We therefore recommended dual voriconazole/caspofungin acetate therapy. Our patient refused to receive dual therapy for financial reasons and potential undesirable liver toxicity. This led to alternative optimal therapy.

Regarding the three reported primary liver aspergillosis cases [10–12], one was successfully treated by caspofungin acetate/amphotericin B dual therapy [10], while the remained two patients died after receiving voriconazole or amphotericin B monotherapy [11,12]. We also noticed one recent study highlighting successful treatment of invasive pulmonary fungal infections with caspofungin acetate single agent first-line therapy in hematological immunocompromised patients [24]. Therefore, caspofungin acetate monotherapy was considered, in view of its broad-spectrum activity and good tolerance. One course of caspofungin acetate first-line therapy (70 mg/day loading dose on day 1 and 50 mg/day maintaining dose from day 2 to day 10) improved the clinical symptoms. A further single course of consolidation therapy produced a favorable response in terms of radiological status. The exact duration of antifungal treatment has not yet been defined, but is likely to be necessary indefinitely. Our patient underwent caspofungin acetate treatment for six months without complications. Complete resolution was achieved clinically and radiographically. To date, six years have passed and the patient remains clinically and radiologically stable.

## 4. Experimental Methods

Direct microscopic examination showed many branched septate hyphae in the liver aspirates on a KOH slide. For fungal culture and isolation, unfixed samples were cultured on routine plate and liquid media, including Sabouraud’s agar. Mycological colonies were grown on Czapek-Dox agar (3.0 g NaNO_3_, 1.0 g KH_2_PO_3_, 0.5 g MgSO_4_, 0.5 g KCl, 0.01 g FeSO_4_, 30.0 g dextrose and 15.0 g agar in 1000 mL H_2_O).

## 5. Conclusions

Collectively, liver involvement of *A. fumigatus* infection is rare, but should not be ignored, especially in non-immunocompromised hosts. Management plans, including surgical resection and antifungal therapy, should be made after systemic examination and evaluation. Caspofungin acetate single agent first-line therapy is recommended for the treatment plans of multiple liver aspergilloma.

## Figures and Tables

**Table 1:** Clinical profiles of liver aspergillosis and therapeutic outcomes in the past decade.

Age	Gender	Underlying disease	Immune status	Involvement	Diagnosis	Treatment	Course	Reference
11	Male	AML	Compromised	Liver	Biopsy	Caspofungin Amphotericin B	Cured	10
53	Female	NHL	Compromised	Liver	Biopsy	Voriconazole	Died	11
66	Male	Aplastic anemia	Compromised	Liver	Biopsy	Amphotericin B	Died	12*
55	Female	Aplastic anemia	Non-compromised	Liver	Biopsy	Caspofungin	Cured	Our case

AML: acute myeloblastic leukemia; NHL: non-Hodgkin’s lymphoma;

* The aplastic anemia patient presented in reference 12 received extensive immunodepressants and was an under immunocompromised condition. Our present aplastic anemia patient did not receive such medications and she was in a relatively non-immunocompromised condition.
